# Electrically tunable terahertz metamaterials with embedded large-area transparent thin-film transistor arrays

**DOI:** 10.1038/srep23486

**Published:** 2016-03-22

**Authors:** Wei-Zong Xu, Fang-Fang Ren, Jiandong Ye, Hai Lu, Lanju Liang, Xiaoming Huang, Mingkai Liu, Ilya V. Shadrivov, David A. Powell, Guang Yu, Biaobing Jin, Rong Zhang, Youdou Zheng, Hark Hoe Tan, Chennupati Jagadish

**Affiliations:** 1 School of Electronic Science and Engineering, Nanjing University, Nanjing 210093, China; 2 Department of Electronic Materials Engineering, Research School of Physics and Engineering, The Australian National University, Acton, ACT 2601, Australia; 3 Collaborative Innovation Center of Advanced Microstructures, Nanjing University, Nanjing 210093, China; 4 Nonlinear Physics Centre, Research School of Physics and Engineering, The Australian National University, Acton, ACT 2601, Australia

## Abstract

Engineering metamaterials with tunable resonances are of great importance for improving the functionality and flexibility of terahertz (THz) systems. An ongoing challenge in THz science and technology is to create large-area active metamaterials as building blocks to enable efficient and precise control of THz signals. Here, an active metamaterial device based on enhancement-mode transparent amorphous oxide thin-film transistor arrays for THz modulation is demonstrated. Analytical modelling based on full-wave techniques and multipole theory exhibits excellent consistent with the experimental observations and reveals that the intrinsic resonance mode at 0.75 THz is dominated by an electric response. The resonant behavior can be effectively tuned by controlling the channel conductivity through an external bias. Such metal/oxide thin-film transistor based controllable metamaterials are energy saving, low cost, large area and ready for mass-production, which are expected to be widely used in future THz imaging, sensing, communications and other applications.

During the past few decades, terahertz (THz) science and technology have achieved tremendous progress because of their importance in the medical, security and manufacturing sectors. In the search for materials to overcome the accessibility difficulties in the THz gap (0.1–10 THz), a class of composite artificial materials termed electromagnetic metamaterials has emerged, in which the resonance can be modified by light, electrical field, magnetic field, temperature, or mechanical strain^1^,^2^,^3^,^4^. Given such external stimulus tend to affect their response, the metamaterial can be dynamically tuned to enable modulation of THz radiation in amplitude, phase, polarization or frequency as it propagates through the system.

Amongst the various ways of accomplishing active tunable THz materials, one popular technique is by taking the advantage of large doping density and high electron mobility in single crystalline semiconductors (e.g., Si, GaAs, graphene)^4^,^5^,^6^. Upon carrier depletion, dynamically switchable THz metamaterial devices have been achieved in Schottky diodes fabricated on semi insulating-GaAs substrates^3^. Constituent resonators can also be switched via external optical excitation of free charge carriers in Si islands or capacitor plates^4^. For ultrafast speed, high-mobility two-dimensional electron gas (2DEG) and graphene were utilized by integration of transistors at the metamaterial unit cell level^5^,^6^,^7^,^8^. However, despite their attractive properties, tunable metamaterials or modulators based on these materials are unsuitable for large area fabrication, and actually pose more stringent requirement on complex growth process as well as high-cost substrates.

Amorphous oxide semiconductors (AOS) typified by In-Ga-Zn-O (IGZO) exhibit a unique combination of high electron mobility (10–50 cm^2^/Vs), high optical transparency and low-temperature processing requirements^9^,^10^,^11^. The mass-production of AOS-based thin-film transistor (TFT) arrays with excellent uniformity can be realized at room temperature on large size substrates through a physical vapor deposition technique known as sputtering. These AOS transistors have been utilized as drive modules of backplanes for the production of high-speed switching devices used in high-motion-speed sensors/displays with ultra-high definition, such as active-matrix liquid crystal displays and active-matrix organic-emitting-diode displays^12^,^13^. As compared to transistors based on 2DEG or graphene materials, the amorphous-IGZO (*a*-IGZO) TFTs exhibit extremely low off-state current (<10^−12^ A), high on-to-off ratio (>10^9^), small subthreshold swing (<0.2 V/dec) and high device yield (close to 100%) over a large area^14^,^15^. Under excitation conditions, the IGZO channel conductivity can be widely tuned up to ~5 × 10^2^ S m^−1^ 10. All these characteristics demonstrate that IGZO TFT arrays have great potential in the application of array-distributed active metamaterial devices with their advantages in energy saving, low cost and high yields. In addition, *a*-IGZO TFTs exhibit excellent light insensitivity owing to the wide bandgap of over 3 eV. Thus great advantages can be obtained in applicability as compared to Si, GaAs and graphene, since THz modulators based on these narrow bandgap semiconductors may require light shielding to eliminate photoconduction-related effects. The high operation stability of *a*-IGZO TFT also ensures its tolerance to large temperature variations and severe environment^9^. Therefore, it is believed that the incorporation of AOS into metamaterials will offer a solution for the mass-production of stable, uniform and low-cost THz metadevices, which may expand the horizon of oxide electronics into the applications of THz modulation and imaging.

In this work, an electrically tunable metamaterial device with monolithic integration of *a*-IGZO TFTs has been designed, simulated and experimentally demonstrated. The metamaterial resonances define the absorption window in transmission spectrum, and the absorption depth can be modulated by tuning the *a*-IGZO conductivity via external electrical bias, which suggests a promising device capable of efficient real-time control and manipulation of THz radiation. The spectral response and modulation performance of the devices are well reproduced by numerical analysis.

## Results

### Design of tunable metamaterials with TFT unit cells

**Figure 1:**
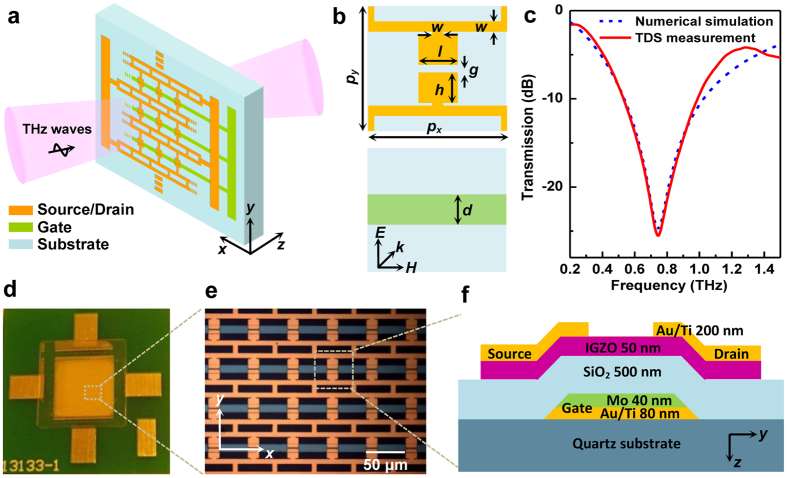
Electrically controlled THz metamaterial with *a*-IGZO TFTs. (**a**) Experimental schematic of the metamaterial device. (**b**) Schematic of two metal layers in one unit cell. (**c**) Simulated and measured transmission spectra of the metamaterials without an applied bias. (**d**) Photograph of a fully fabricated device. (**e**) Close-up view of the device. (**f**) Schematic showing the cross section of the *a*-IGZO TFT.

Figure 1a presents a schematic illustration of our electrically controllable metamaterial, which consists of two metal layers (Fig. 1b), separated by a thin dielectric spacer. The top metal layer is patterned to form a two-dimensional (2D) array of electric resonators, which are wired to an external circuit via on-chip connections, naturally functioning as drain and source electrodes for transistors in each unit cell. The array of parallel wires with uniform spacing formed in the second metal layer are used for connecting all the gates within the same row. This differs from the usual design^3^, as all the metamaterial elements in this work are also connected along *y* direction, strong coupling between individual resonators occurs and would have significant influence on the electromagnetic properties of resonance. The metamaterial geometry (Fig. 1b) used is optimized as follows: the period *p*_*x*_ = *p*_*y*_ = 50 μm, the line width *w* = 4 μm, the dielectric gap *g* = 3 μm, the capacitor dimensions *h* = 12.5 μm, *l* = 14 μm, and the gate width *d* = 11 μm. When the polarization of incident THz electric field is perpendicular to the split gap (i.e., 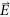//*y*-axis), an intrinsic resonance mode can be observed as shown in Fig. 1c. In our experiments, the THz metamaterials cover an area of 1 cm × 1 cm. Photographs of the fabricated device are shown in Fig. 1d,e with the schematic cross section of a unit cell depicted in Fig. 1f. The thickness of *a*-IGZO active layer and SiO_2_ insulating layer is 550 nm in total. When a positive gate bias is applied to the transistor arrays, the channel currents will flow in the active layer, which has a tunable conductivity. In this manner, the THz electric near-field is enhanced in the gap of the capacitors and the electromagnetic properties of the resonators can be dynamically tailored.

**Figure 2:**
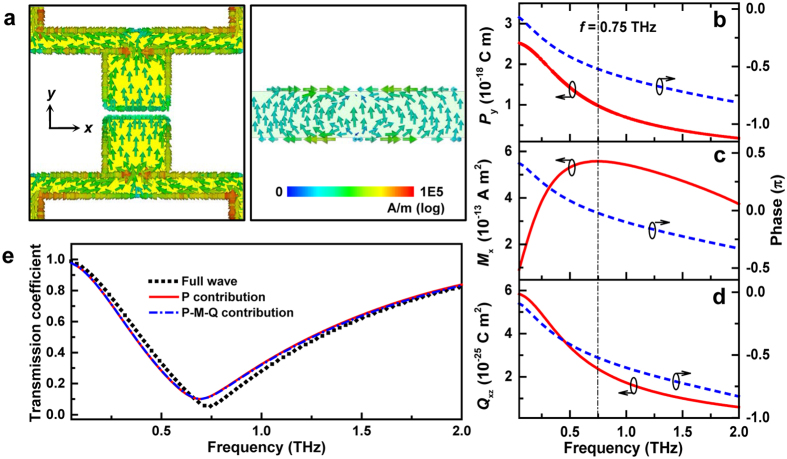
Theoretical calculation of the resonance mode illustrating the underlying mechanism. (**a**) Surface currents distribution (peak value) at 0.75 THz in one unit cell for initial state (i.e., gate bias is zero). The colored arrows indicate the direction and density of surface currents. (**b–d**) The electric dipole, magnetic dipole and electric quadrupole moments (*P*_*y*_, *M*_*x*_, *Q*_*xz*_) as functions of frequency, including amplitude and phase. (**e**) The transmission coefficient (amplitude) calculated by full-wave simulation or by fitting the multipolar expansion model, i.e., Eq. (4).

To illustrate the underlying mechanism responsible for the resonant behavior at 0.75 THz, the surface current distribution profile in one unit cell is plotted by using a commercially available CST Microwave Studio on a logarithmic scale shown in Fig. 2a. The colored arrows visually represent the vector nature of the surface currents, including the intensity and direction. Two main features should be pointed out. First, there is significant current flowing along the connecting metal wires in this particular metamaterial design, which confirms that the resonance mode is strongly affected by these connecting wires, and consequently the resonance frequency red-shifts from 2.1 to 0.75 THz when adding these connections (see Supplementary Fig. S1). Second, the net currents flowing through the two metal layers are almost in parallel with each other, which indicates an electric dipole-type response occurring at resonance frequency. To numerically verify the nature of the resonance, a common approach is to retrieve the effective permittivity and permeability from the S parameters. However, the results have non-physical features for such a 2D metamaterial with a finite thickness^16^. Instead, the macroscopic electric and magnetic polarization as function of frequency was calculated, and the multipoles that dominate the scattering response of resonators were consequently identified through the use of multipolar expansion^17^,^18^. By taking the following definitions, the electric dipole 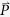, quadrupole 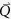 and magnetic dipole moments 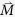 over a unit cell in terms of surface current density 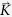 were calculated:


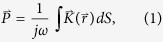



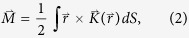



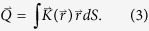


With reference to Fig. 2a, the dominant electric dipole moment will be in the *y* direction, while the magnetic moment will be oriented along the *x* direction. Due to spatial asymmetry of the structure in the *z* direction, a non-zero electric quadrupole moment should be at least considered since its contribution to the forward scattering may be comparable to the magnetic dipole moment. In Fig. 2b–d, the amplitude and phase of these components in the volume of a unit cell is plotted. Here the phase is calculated relative to the incident wave phase at the top surface of the sample, with the complex phase factor, exp[*j*(*ωt* − *kz*)]. The coordinate origin is chosen at the middle between these two metal layers (see Supplementary Fig. S2). Based on classical multipole theory, the electromagnetic properties of a scatterer, e.g., the transmitted field, can be modeled as follows^19^,^20^


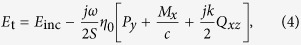


here *E*_inc_ is the incident field, 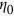 the free-space impedance, *k* the free-space wave number and *S* the unit cell area in the square array. The transmission coefficient can thus be calculated by *E*_t_/*E*_inc_ as shown in Fig. 2e. It is found that the transmission induced by the electric dipole (*P*_*y*_) exhibits a good fit to the full-wave simulation result, and the change of the curve due to the inclusion of *M*_*x*_ and *Q*_*xz*_ is negligible. It proves that the electric dipole moment provides the dominant contribution to the radiation. Furthermore, these results show that the system exhibits a highly-damped electric-dipole response, which is fairly broadband in nature. The resonant dip in transmission observed in Fig. 2e is not due to the enhancement of current at the resonant frequency. Instead, it comes from the re-radiated dipole field being out of phase with the incident field, whilst having comparable magnitude. This can be seen from Eq. (4) by considering the case when the phase shift of *P*_*y*_ equals to *π*/2, corresponding to the vertical line in Fig. 2b. On the basis of the above analysis, it is believed that the resonance mode at 0.75 THz in this hybrid structure has an almost purely electric dipole type response.

**Figure 3:**
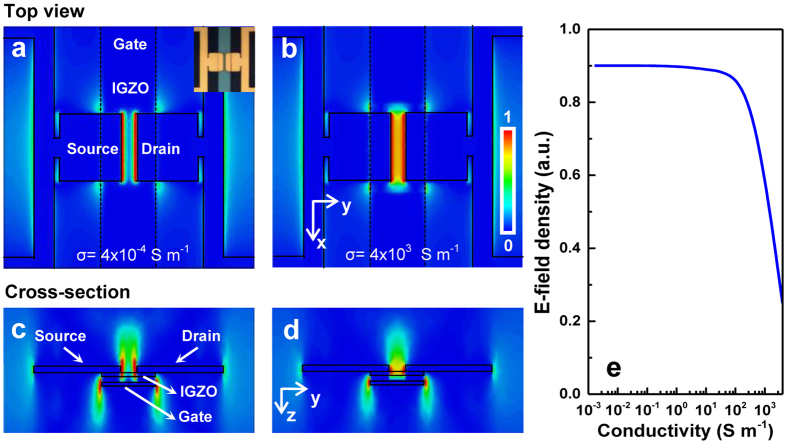
Simulated THz-electric field in the hybrid metamaterial. (**a,b**) Normalized electric field (|*E*_*y*_| component) distribution in the middle plane of channel layer within one metamaterial unit cell for two different IGZO conductivities (4 × 10^−4^ and 4 × 10^3^ S m^−1^). (**c,d**) The corresponding cross-sectional views of |*E*_*y*_| distribution shown in (**a,b**). (**e**) The calculated THz *E*-field density in *a*-IGZO layer in a unit cell area, which is integrated in a volume of 11 × 18 × 0.05 μm^3^ around the split gap.

The tuning performance of the metamaterial can be numerically predicted by investigating the relationship between the metamaterial transmission resonance and the gate bias. The latter is accounted for by introducing gate bias-dependent conductivity *σ*_*a*-IGZO_ in the *a*-IGZO channel. Figure 3a,b show the normalized |*E*_*y*_| component distribution in the middle plane of the channel layer within one unit cell when the conductivity of the *a*-IGZO thin film under the split gaps is increased from 4 × 10^−4^ to 4 × 10^3^ S m^−1^. These values are employed in CST simulations based on *a*-IGZO TFT I − V characteristics that have been previously reported in^14^,^15^. Their corresponding cross-section views are shown in Fig. 3c,d. Without an external bias, the *a*-IGZO channel films are insulating and dielectric due to the complete depletion in the off-state. Under a positive bias, for example, when the conductivity is increased to 4 × 10^3^ S m^−1^, the injected carriers within the IGZO area result in a shrinkage of effective gap between source and drain as descripted in Fig. 3b,d. To explore the physical origin of the tunable behavior of resonance mode, the THz-electric-field intensity within the channel layer was calculated by a volume integral to study the effect of conductivity on the resonance strength (see Fig. 3e). Upon positive bias, the conductivity of *a*-IGZO area is expected to increase and the THz-electric field expands into the split gaps. However, the overall THz energy accumulated in the vicinity of the gaps is severely suppressed due to the reduced impedance of the gap^21^. This could be easily understood that the increased conductivity of the gap leads to the reduced voltage drop across the gap, and therefore the decrease of the THz electric field.

### Electrical control of metamaterials

**Figure 4:**
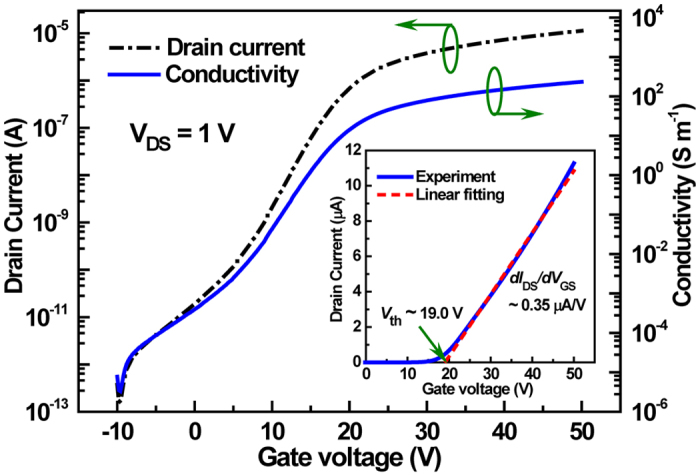
Transfer characteristics of the *a*-IGZO TFTs and the extracted conductivity of the *a*-IGZO channel with various gate voltages. The inset shows a linear fitting curve to the drain current.

Figure 4 illustrates the typical transfer characteristics and channel conductivity of *a*-IGZO TFTs with a source-drain voltage of 1 V. From the linear region, the threshold voltage (*V*_th_) and mobility (*μ*_fe_) can be extracted as shown in the Method section. The straight line in the inset of Fig. 4 represents the best linear fit between 90 to 10% of the maximum *I*_D_ (at *V*_GS_ = 45 V) and *V*_th_ and *μ*_fe_ are determined to be 19.0 V and 7.9 cm^2^/Vs, respectively. Here the comparatively larger *V*_th_ is mainly attributed to the use of thicker gate insulator layer as compared to our previous design^15^. The conductivity of *a*-IGZO channel displays a clear increase as the gate voltage sweeps from off-state to on-state with an on-to-off ratio of approximately 10^7^, revealing the modulating ability of gate bias on the conductivity of *a*-IGZO and thereby the resonant properties of metamaterials. It should be noted that, based on a separate contact resistance measurement, the contact resistance between the source/drain and the *a*-IGZO film is much smaller than the channel resistance^15^. Therefore, the measured I − V characteristics should mainly reflect the carrier transport properties of *a*-IGZO channel.

**Figure 5:**
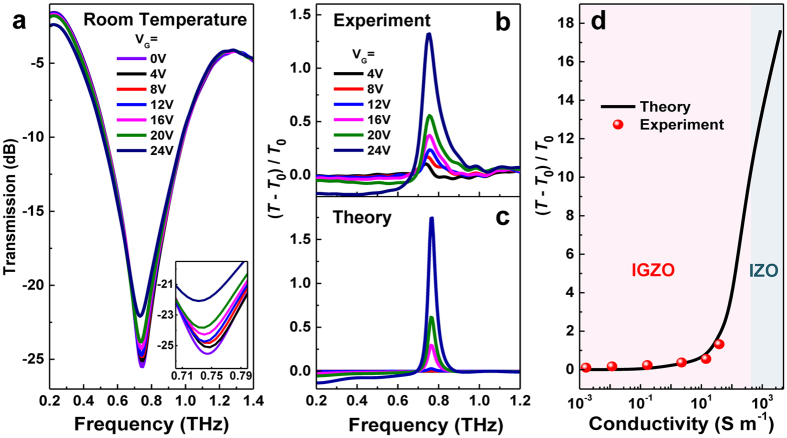
Active control of the THz waves. (**a**) Transmission spectra at different gate bias. Inset shows a zoom-in view around the resonance. (**b**) Measured and (**c**) simulated differential transmission with sweeping gate voltages. (**d**) The relationship between differential transmission and conductivity extending up to 4 × 10^3^ S m^−1^.

Room-temperature THz transmission measurements were performed with a THz time-domain spectroscopy (THz-TDS) system in a nitrogen-purged environment. The incident THz radiation was normal to the planar metamaterial structure and the THz electric field was perpendicular to the split gaps so as to couple to the capacitive element. Figure 5a shows the THz transmission spectra of the metamaterial structure when applying different positive bias (*V*_G_) to the individual transistor unit ranging from 0 to 24 V. With drain/source electrodes grounded, the resonant behavior of the device is strongly dependent on the gate bias. At zero gate bias, the metamaterial resonance shows significant frequency dependence near the resonant frequency of 0.75 THz, as the *a*-IGZO material exhibits dielectric properties with ultrahigh resistance and thus resonance is established. Because these transistors are working in an enhanced mode, the *a*-IGZO underneath layer has no effect on the resonance characteristics of metamaterials (e.g., the overall transmission) as the devices are in off-state. The low energy consumption of these transistors in the off-state is an advantage over THz modulators based on graphene or 2DEG which worked in a depletion mode. By increasing the forward bias, free electrons are induced and accumulated at the interface between the oxide dielectric (SiO_2_) and IGZO channel, which thereby gradually shorts the capacitive split gap, and the resonance begins to diminish. As the bias is increased to 24 V, a 4-dB relative change of transmission in intensity is observed while the resonant frequency exhibits a negligible red-shift (about 15 GHz). The tunability of THz amplitude can be controlled precisely by an external bias, making this device a reasonably efficient THz modulator in this frequency region.

To clarify the tuning ability of the THz metamaterials, the differential transmission defined as *D* = (*T* − *T*_0_)/*T*_0_ is plotted in Fig. 5b, where *T* is the transmitivity with *T*_0_ corresponding to that at zero gate bias. As the bias is varied from 0 to 24 V, the conductivity of IGZO is increased from 4 × 10^−4^ to 40 S m^−1^, and a gradual enhancement of differential transmission can be observed, which verifies the extension of electric field into the capacitor gaps as described in Fig. 3. At *V*_G_ = 24 V, the differential transmission of *D* = 1.3 is obtained, which is ~15 times the value at *V*_G_ = 4 V. Comparing the results from numerical simulation (Fig. 5c) with the experimental data, excellent agreement is exhibited. The relationship between differential transmission and conductivity up to 4 × 10^3^ S m^−1^ has also been theoretically extended as shown in Fig. 5d. The results show that our experimental observations fit the theoretical curve fairly well for the range of conductivity investigated here (up to 40 S m^−1^). To achieve very high conductivities, one can increase the In:Ga ratio in the target for the deposition of IGZO active layer^10^,^22^,^23^, since indium content plays a key role in the enhanced electrical performance. In-Zn-O (IZO), whose channel conductivity can be tuned up to 5 × 10^4^ S m^−1^ 10,24, could be taken as an extreme case that has no gallium content. In such a scheme, the properties of metamaterial resonances can be greatly tuned both in amplitude and frequency, and further improvement of modulation depth can also be expected. Thus, these conducting oxide TFTs provide an alternative platform to realize efficient manipulation of THz signals.

### Dynamic characteristics

**Figure 6:**
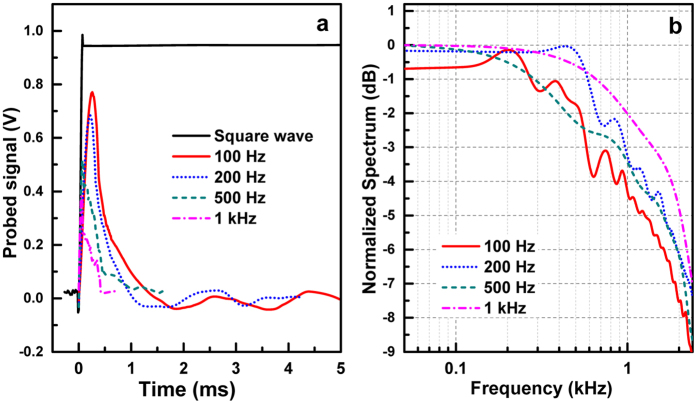
Measured modulation speed. (**a**) Temporal response of the THz-metamaterial device under a rectangular AC gate bias alternating between 0 and 20 V. (**b**) Fourier transform of the temporal response.

The temporal response of the *a*-IGZO TFT based metamaterial device has been measured by applying a rectangular AC gate bias alternating between 0 and 20 V. The temporal response signal corresponding to the charging process was collected at different modulation frequencies of 100, 200, 500 and 1000 Hz. As illustrated in the response waveform to the square modulation voltage (see Fig. 6a), the current signal rises quickly due to the excited carriers in *a*-IGZO channel and then falls back to zero indicating that a new equilibrium state of carrier distribution has been established^25^. The FWHM value of the modulator is measured and remains almost unchanged between 220 and 350 μs. By Fourier transforming the time-domain data, the 3-dB bandwidth of ~1 kHz can be obtained as plotted in Fig. 6b. In our measurement setup, the temporal response results are determined by two physical events. One is the carrier charging process in the *a*-IGZO channel, which is mainly limited by the resistance-capacitance (RC) delay and charge trapping effects at defects (i.e., oxygen vacancies)^26^. The other event is the interaction between incident THz signal and metamaterial resonators. In contrast, time delay associated with the electromagnetic radiation response is negligible and charging time is therefore examined in this work to evaluate the tuning speed of the active metamaterial. To estimate the device RC constant, a resistance (*R*_on_) of 1.57 MΩ is used, which is derived from the measured conductivity as shown in Fig. 4. A single pixel TFT capacitance (*C*_pix_) of 6.8 × 10^−15^ F is calculated using an SiO_2_ thickness of 500 nm and an effective plate area of 11 × 14 μm^2^. Since the modulator contains 200 × 200 TFT pixels, the RC time constant is about 425 μs, in good agreement with the directly measured response time. It can be speculated that a larger area of the metamaterial array will create a larger overall device capacitance, yielding a further limited cut-off modulation frequency. However, together with the material optimization for higher-mobility *a*-IGZO channels, the improved device design by less overlapping between the gate-to-source and drain areas would be expected to shorten the RC delay for higher modulation frequency. On the other hand, based on our previous work^15^, the trapping/de-trapping-related charging time in *a*-IGZO channel can be estimated as fast as 1 μs. Therefore, the speed of our TFT-based metadevice should be dominantly limited by its RC delay.

## Discussion

As indicated by the tunability performance in Fig. 5, our experimental observations fit the theoretical curve very well for the investigated conductivity range (up to 40 S m^−1^). An enhanced differential transmission would be expected if the THz modulators are based on IGZO TFTs with higher channel conductivities. As indicated in literature works^10^,^22^,^23^, an increase in indium composition can improve the electrical performance of IGZO TFTs, because the heavy metal indium cations share electrons via spatially spread 5s orbitals with isotropic shape and act as electron pathways contributing to an enhanced carrier mobility of TFTs. On the other hand, Ga ions are important in forming strong chemical bonds with oxygen to reduce background carriers and off-state leakage current, thus promising energy saving and large-area fabrication^9^,^10^,^22^. The further optimization of sputtering condition and justification of In:Ga ratio are therefore necessary to balance the conductivity and energy consumption of IGZO TFTs for THz modulation applications. In addition, it should be reminded that the carriers in *a*-IGZO or IZO materials mainly originate from oxygen vacancies within the oxide channel^10^,^22^. As the extreme case without gallium content, the large tunable range of conductivity in IZO may indeed enhance the efficiency of THz modulation. However, it may also sacrifice the modulation speed of the device as a result of the enhanced trap-related charging time. This phenomenon, in turn, provides an effective path to investigate the carrier dynamics, charge trapping and de-trapping in defects within oxide devices by THz temporal response characterization.

In summary, an electrically tunable metamaterial has been experimentally demonstrated by hybridizing *a*-IGZO TFTs into unit cells. Numerical analysis based on the TDS measurement verifies that this novel resonance mode at 0.75 THz is mainly attributed to an electric-dipole response to the external THz field, and its properties can be effectively controlled by electrically tuning the conductivity of the active IGZO layer. Experimentally, a 4-dB relative intensity change can be observed at a forward gate bias of 24 V. Despite the large device area, it still has a cut-off frequency of ~1 kHz. Considering the attractive properties of *a*-IGZO as well as the high performance of oxide TFTs, devices based on transparent oxide TFTs monolithically integrated with a metamaterial unit cell level might present a new platform for exploring stable, uniform and low-cost modulators in THz and other frequency range.

## Methods

### Device fabrication

The whole metamaterial structure hybridized with IGZO TFTs was fabricated on a 500 μm-thick quartz substrate which has a relative permittivity of 3.75 (see Fig. 1d). The IGZO TFTs are in a back-gated configuration and the bottom gate electrode composed of Ti/Au/Mo (30/50/40 nm) was deposited by e-beam evaporation and further patterned with the lift-off technique. A thick SiO_2_ gate insulator of 500 nm was then deposited by plasma-enhanced chemical vapor deposition at 300 °C. It was found that the increase of dielectric thickness can effectively minimize the implication of back gate metals electrode on the resonant properties of the final metamaterial device. Subsequently, a 50 nm *a*-IGZO active layer was deposited by sputtering at room temperature in an oxygen partial pressure of 0.5 Pa and the used target has the composition of In_2_O_3_:Ga_2_O_3_:ZnO = 1:1:1 in mole ratio. The 2D array of electric resonators was defined directly on the *a*-IGZO layer with photolithographic technique and e-beam evaporation of Ti/Au (30/170 nm). Finally, contact window of gate electrode was selectively opened by wet etching. A following post-annealing process was carried out in air at 300 °C for 60 minutes to enhance the performance and operation stability of *a*-IGZO TFTs. The fabrication process is similar to our previous work on *a*-IGZO based inverter^15^.

### Numerical simulations

Full-wave simulations on the above metamaterials were performed using the commercial software package CST Microwave Studio. In the simulation, the quartz substrate was treated as a loss free dielectric and Au as a lossy metal with an assumed conductivity of 4.56 × 10^7^ S m^−1^. To approximate the implications on the resonance from modification of *a-*IGZO channel, the *a-*IGZO is simulated with the permittivity infinity *ε*_*a*-IGZO_ of ~4^27^ and a gate bias-dependent conductivity *σ*_*a*-IGZO_. To study the transmission properties and E-field intensity distribution in the metamaterial, frequency-domain solver is used to solve the integral formulation of Maxwell equations by adopting the Finite Integration Technique with Floquet Boundary conditions. For a high degree of accuracy, the tetrahedral meshing is applied with special care into the metal structures.

### TFT characterization

The electrical characterization of the *a*-IGZO based TFTs was carried out with a Keithley 2636 system. For measurement of the transfer characteristics, the source electrode was grounded and *V*_DS_ was set to 1 V, while the drain current was measured with gate bias sweeping from pinch-off state to on-state. The resulting curve is shown in Fig. 4, revealing the modulating ability of gate bias on the conductivity of the *a*-IGZO channel. As well known, the standard MOSFET equation in linear region can be simplified under the condition of *V*_DS_ ≪ (*V*_GS_ − *V*_th_) to


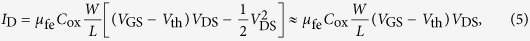


where *C*_ox_ is the gate insulator capacitance per unit area, *W* and *L* are TFT channel width and length, respectively. Therefore, *V*_th_ and *μ*_fe_ can be extracted from the linear fit of Eq. (5).

### THz-TDS characterization

The THz transmission measurements were performed with a THz-TDS system (Advantest TAS7500SP) in a nitrogen-purged environment (see Supplementary Fig. S3). A DC Power Supply (Tektronix, PWS2326-SC) was used to apply a series of constant voltage bias between gate and source/drain electrodes of IGZO TFTs to realize *in-situ* control of channel conductivity. For each voltage step, the transmission spectrum was measured using the THz-TDS system with two femtosecond lasers utilized instead of a mechanical delay stage. The THz pulse, obtained from a THz emitter irradiated by a femtosecond laser pulse (with a wavelength of 1560 nm, a pulse width of about 45 fs and a repetition rate of 50 MHz), was radiated perpendicular to the sample via a set of off-axis parabolic mirrors. Then, the transmitted THz signal was focused by another set of off-set parabolic mirrors on a photoconductive antenna-based THz detector, in which the pulse component was synchronized with another femtosecond laser pulse, of which the time delay, *t* (ps), was changed stepwise at a constant interval of 2 fs. The detected electromagnetic waves were processed into a voltage, *V* (mV), in a lock-in amplifier. The voltage for each time delay was measured 4096 times and averaged to obtain the waveform *V*(*t*). All transmitted signals collected were then normalized to that obtained from a quartz substrate. The THz-TDS system has a usable bandwidth from 0.2 to 4 THz and a signal-to-noise ratio of over 10,000:1.

### Temporal response measurement

To analyze the dynamic electrical properties of the THz modulator, an arbitrary signal generator (Tektronix, AFG3102C) was utilized to apply a rectangular AC forward gate bias voltage alternating between 0 and 20 V. A two-channel digital oscilloscope (Tektronix, DPO4032) with a time resolution of 0.4 ns was utilized in series to detect the current corresponding to the charging or depletion process of the *a*-IGZO channel. Considering the small series resistance (50 Ω) of the digital oscilloscope, the voltage drop on it is negligible. Therefore, the output of the oscilloscope can be used to examine the temporal response of the THz modulator based on *a*-IGZO TFTs.

## Additional Information

**How to cite this article**: Xu, W.-Z. *et al*. Electrically tunable terahertz metamaterials with embedded large-area transparent thin-film transistor arrays. *Sci. Rep.* **6**, 23486; doi: 10.1038/srep23486 (2016).

## Supplementary Material

Supplementary Information

## References

[b1] Pendry J. B., Schurig D. & Smith D. R. Controlling Electromagnetic Fields. Science 312, 1780 (2006). 16728597 10.1126/science.1125907

[b2] Shen N.-H. . Broadband blueshift tunable metamaterials and dual-band switches. Phys. Rev. B 79, 161102 (2009).

[b3] Chen H.-T. . Active terahertz metamaterial devices. Nature 444, 597 (2006). 17136089 10.1038/nature05343

[b4] Chen H.-T. . Experimental demonstration of frequency-agile terahertz metamaterials. Nat. Photon. 2, 295 (2008).

[b5] Lee S. H. . Switching terahertz waves with gate-controlled active graphene metamaterials. Nat. Mater. 11, 936 (2012). 23023552 10.1038/nmat3433

[b6] Valmorra F. . Low-bias active control of terahertz waves by coupling large-area CVD graphene to a terahertz metamaterial. Nano Lett. 13, 3193 (2013). 23802181 10.1021/nl4012547

[b7] Xu W., Padilla W. J. & Sonkusale S. Loss compensation in metamaterials through embedding of active transistor based negative differential resistance circuits. Opt. Express 20, 22406 (2012). 23037389 10.1364/OE.20.022406

[b8] Shrekenhamer D. . High speed terahertz modulation from metamaterials with embedded high electron mobility transistors. Opt. Express 19, 9968 (2011). 21643254 10.1364/OE.19.009968

[b9] Nomura K. . Room-temperature fabrication of transparent flexible thin-film transistors using amorphous oxide semiconductors. Nature 432, 488 (2004). 15565150 10.1038/nature03090

[b10] Nomura K. . Amorphous oxide semiconductors for high-performance flexible thin-film transistors. Jpn. J. Appl. Phys. 45, 4303 (2006).

[b11] Hsu H.-H., Chang C.-Y., Cheng C.-H., Chiou S.-H. & Huang C.-H. High mobility bilayer metal–oxide thin film transistors using titanium-doped InGaZnO. IEEE Elec. Dev. Lett. 35, 87 (2014).

[b12] Jeong J. K. The status and perspectives of metal oxide thin-film transistors for active matrix flexible displays. Semicond. Sci. Technol. 26, 034008 (2011).

[b13] Ohara H. . 4.0-inch active-matrix organic light-emitting diode display integrated with driver circuits using amorphous In-Ga-Zn-Oxide thin-film transistors with suppressed variation. Jpn. J. Appl. Phys. 49, 03CD02 (2010).

[b14] Sato A. . Amorphous In-Ga-Zn-O coplanar homojunction thin-film transistor. Appl. Phys. Lett. 94, 133502 (2009).

[b15] Huang X. . Large-swing *a*-IGZO inverter with a depletion load induced by laser annealing. IEEE Elec. Dev. Lett. 35, 1034 (2014).

[b16] Simovski C. R. & Tretyakov S. A. Local constitutive parameters of metamaterials from an effective-medium perspective. Phys. Rev. B 75, 195111 (2007).

[b17] Powell D. A., Shadrivov I. V. & Kivshar Y. S. Nonlinear electric metamaterials. Appl. Phys. Lett. 95, 084102 (2009).

[b18] Raab R. E. & de Lange O. L. Multipole Theory in Electromagnetism (Clarendon, Oxford 2005).

[b19] Niemi T., Karilainen A. O. & Tretyakov S. A. Synthesis of polarization transformers. IEEE Trans. antennas propag. 61, 3102 (2013).

[b20] Ra’di Y., Asadchy V. S. & Tretyakov S. A. Tailoring reflections from thin composite metamirrors. IEEE Trans. antennas propag. 62, 3749 (2014).

[b21] Chen H.-T. . A metamaterial solid-state terahertz phase modulator. Nat. Photon. 3, 148 (2009).

[b22] Fortunato E. . Oxide semiconductor thin-film transistors: a review of recent advances. Advan. Mater. 24, 2945 (2012). 22573414 10.1002/adma.201103228

[b23] Kim G. H. . Effect of indium composition on solution-processed nanocrystalline InGaZnO thin film transistors. Appl. Phys. Lett. 94, 233501 (2009).

[b24] Leenheer A. J. . General mobility and carrier concentration relationship in transparent amorphous indium zinc oxide films. Phys. Rev. B 77, 115215 (2008).

[b25] Paul O. . Polarization-independent active metamaterial for high-frequency terahertz modulation. Opt. Express 17, 819 (2009). 19158896 10.1364/oe.17.000819

[b26] Jeon S. . Gated three-terminal device architecture to eliminate persistent photoconductivity in oxide semiconductor photosensor arrays. Nat. Mater. 11, 301 (2012). 22367002 10.1038/nmat3256

[b27] Galca A. C., Socol G. & Craciun V. Optical properties of amorphous-like indium zinc oxide and indium gallium zinc oxide thin films. Thin Solid Films 520, 4722 (2012).

