# Supplementary material for: Electrically tunable terahertz metamaterials with embedded large-area transparent thin-film transistor arrays
Source: Sci Rep. 2016 Mar 22;6:23486. doi: 10.1038/srep23486 (PMC4802223; doi:10.1038/srep23486)
Supplement: Supplementary Information [file srep23486-s1.pdf]

Supplementary Information

Electrically tunable terahertz metamaterials with embedded large-area transparent thin-film transistor arrays

Wei-Zong Xu[1,2,3], Fang-Fang Ren[1,2,3], Jiandong Ye[1,2], Hai Lu[1,3], Lanju Liang[1], Xiaoming Huang[1,3], Mingkai Liu[4], Ilya V. Shadrivov[4], David A. Powell[4], Guang Yu[1,3], Biaobing Jin[1], Rong Zhang[1], Youdou Zheng[1], Hark Hoe Tan[2] & Chennupati Jagadish[2]

[1]*School of Electronic Science and Engineering, Nanjing University, Nanjing 210093, China*

[2]*Department of Electronic Materials Engineering, Research School of Physics and Engineering, The Australian National University, Canberra, ACT 2601, Australia*

[3]*Collaborative Innovation Center of Advanced Microstructures, Nanjing University, Nanjing 210093, China*

[4]*Nonlinear Physics Centre, Research School of Physics and Engineering, The Australian National University, Canberra, ACT 2601, Australia*

Correspondence and requests for materials should be addressed to F.F.R. (ffren@nju.edu.cn) or H. L. (hailu@nju.edu.cn)

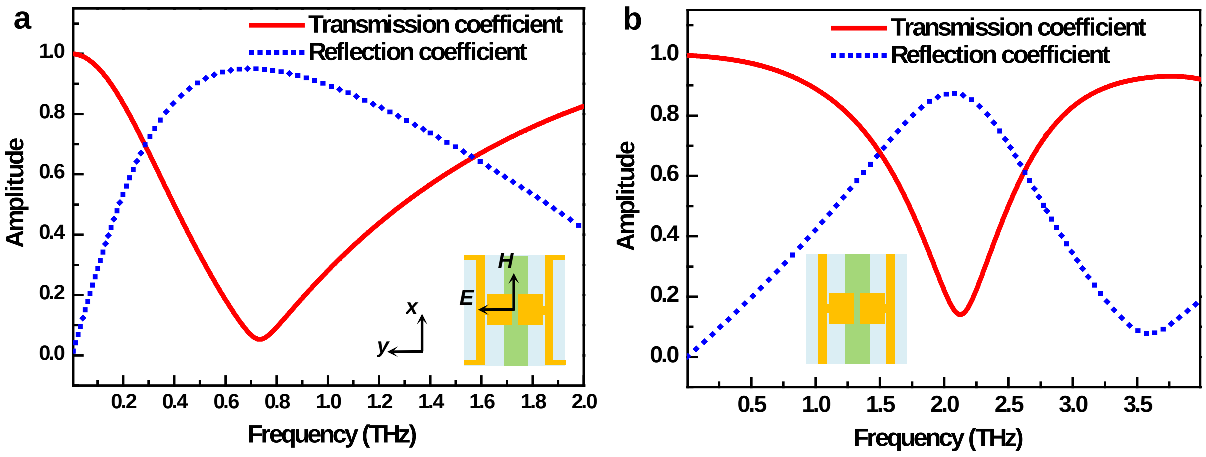

Figure S1. Transmission/reflection coefficient of *a*-IGZO TFT-based metamaterials with (a) connected or (b) unconnected metamaterial design. The resonance frequency red-shifts from 2.1 to 0.75 THz due to the connecting design along *y*-direction in our structure.

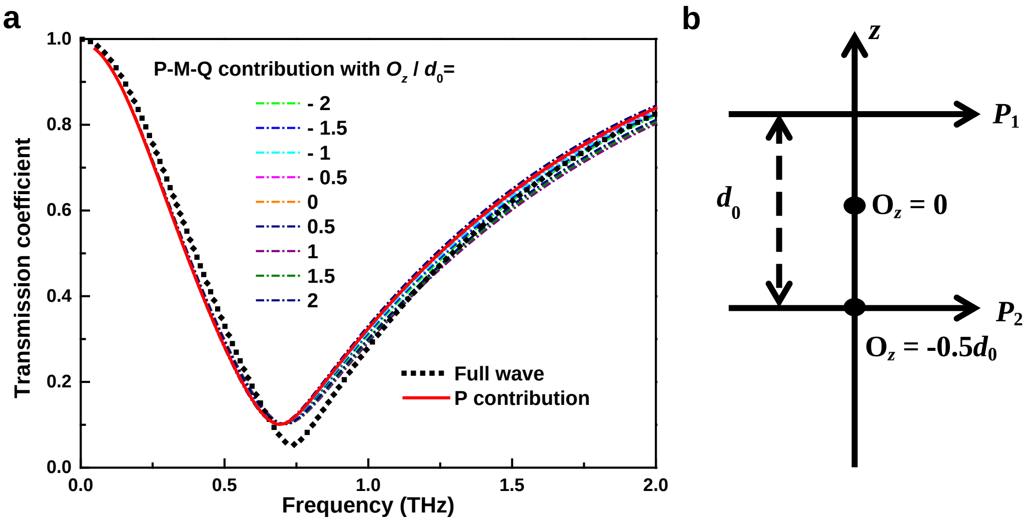

Figure S2. The choice of coordinate origin for calculating magnetic dipole (*M*) and electric quadruple (*Q*) moments. (a) The transmission coefficient (amplitude) calculated by fitting the multipolar expansion model with different choice of coordinate origin O_z. (b) Schematic of the coordinate origin. Here P_1 and P_2 represent the electric dipole moments of source/drain layer and gate layer, respectively. The distance between these two layers is indicated as d_0. We found that the different choice of coordinate origin O_z for calculating *M* and *Q* results only in a small difference to the transmitted field, while the electric dipole moment (*P*) unambiguously remains dominant to the scattered radiation. Therefore, we simply show the results in Figs. 2c-2e by choosing the origin at the middle of these two metal layers.

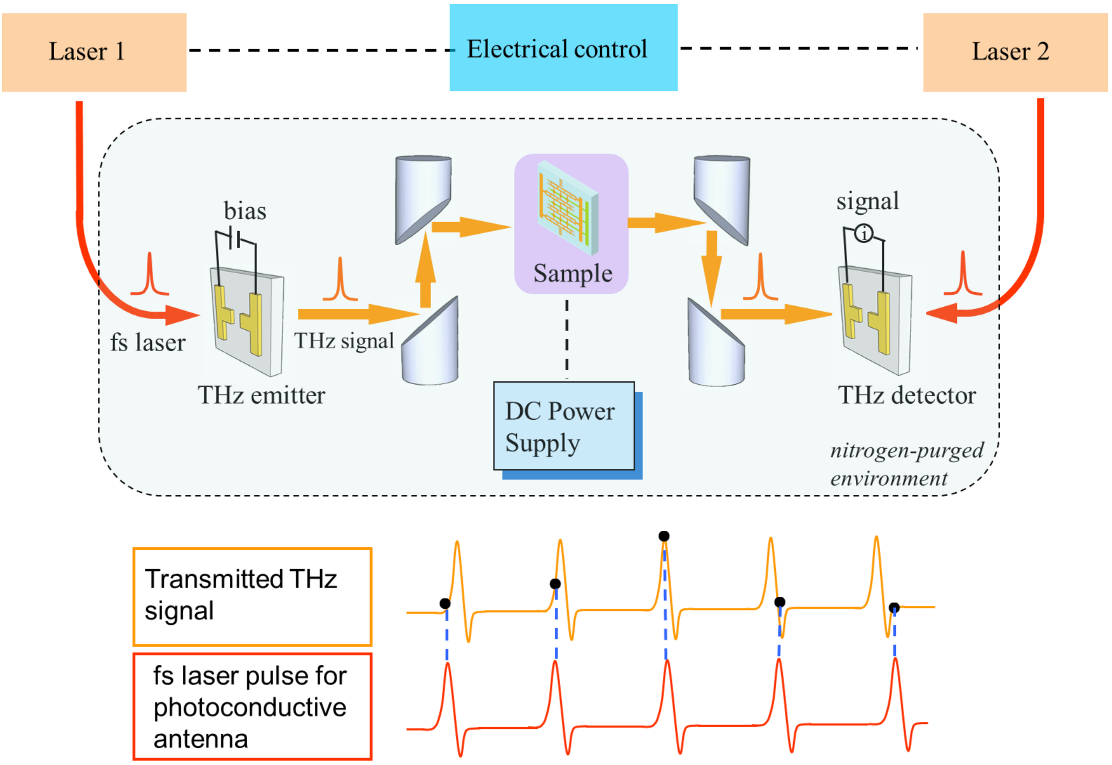

Figure S3. Experimental setup for characterizing the tunable THz metamaterial.
